# First identification of human infection with *Erysipelothrix Piscisicarius* by metagenomic next-generation sequencing

**DOI:** 10.1080/22221751.2022.2140614

**Published:** 2022-11-11

**Authors:** Weifeng Huang, Dan Han, Qingqing Cai, Xiaoli Yi, Jin Tang, Yuan Fang, Yihan Lu

**Affiliations:** a Department of Intensive Care Medicine, Shanghai Sixth People's Hospital Affiliated to Shanghai Jiao Tong University School of Medicine, Shanghai, People’s Republic of China; b Department of Emergency Internal Medicine, Shuguang Hospital, Shanghai University of Traditional Chinese Medicine, Shanghai, People’s Republic of China; c Genoxor Medical Science and Technology Inc., Shanghai, People’s Republic of China; d Department of Clinical Laboratory, Shanghai Sixth People's Hospital Affiliated to Shanghai Jiao Tong University School of Medicine, Shanghai, People’s Republic of China; e Department of Epidemiology, Ministry of Education Key Laboratory of Public Health Safety, School of Public Health, Fudan University, Shanghai, People’s Republic of China

To the editor: *Erysipelothrix* genus, a kind of gram-positive facultative anaerobes, is known to cause Erysipelas in pigs and Erysipeloid in humans [1]. Currently, *Erysipelothrix* genus is composed of five species, including *Erysipelothrix rhusiopathiae*, *Erysipelothrix tonsillarum*, *Erysipelothrix inopinata*, *Erysipelothrix larvae*, and *Erysipelothrix piscisicarius*[2]. *E. piscisicarius* is a novel pathogen in fish aquaculture, causing orofacial ulceration and necrosis, necrotizing dermatitis, myositis, and cellulitis in fish [3]. So far, *E. piscisicarius* infection has not been documented in humans. Herein, we reported a case of *E. piscisicarius* infection in a woman detected by metagenomics next-generation sequencing (mNGS) and confirmed by whole genome sequencing (WGS), and it is the first case of *E. piscisicarius* infection in humans.

A 72-year-old female patient was admitted to our hospital with a complaint of high fever and chills of unknown origin (Day 1). In the emergency department, she reached a body temperature of 40.2°C, combined with headache, vertigo, anhelation, lags in response, drowsiness, and urine retention. Physical examination showed pulse of 125 bpm, 39 breaths/min, and blood pressure of 89/45 mmHg. After blood routine testing (Table S1), the patient was transferred to EICU for further treatment. Piperacillin-tazobactam (4.5 g q8h) was empirically administrated for the patient, combined with symptomatic treatment like reducing sputum and nutrition support. The initial symptoms of the patient had been relieved, including dizziness, headache, and anhelation (Day 2). However, she remained a fever with a cold, with a maximum body temperature of 38.0°C. With active treatment, the patient's body temperature gradually returned to normal (Day 3).

Evaluations to determine the microbiological cause of fever included blood culture and mNGS. The detailed methods were shown in the Technical Appendices. The mNGS achieved 20,849,484 reads; after quality control processing, we obtained 19,247,085 clean reads for the downstream analysis. Nine reads of *E. piscisicarius* were detected in the peripheral blood by mNGS (Day 4). In contrast, the blood culture result showed an infection of *E. rhusiopathiae*, verified by the Matrix-Assisted Laser Desorption Ionization–Time of Flight Mass Spectrometry subsequently (Figure S1). Due to this inconsistency, the cultured microorganisms were sent for WGS. The acquired sequence matched the *E. piscisicarius* whole genome at a coverage rate of 91.2% (Figure 1A), confirming the mNGS result. Just then, a red and swollen mass (1 × 0.5 cm) at the fingertip of the patient's left thumb was noted (Figure 1B). The patient complained that the finger had been punctured when washing shrimps, which was later identified as *Penaeus vannamei*, three days before the onset of the disease. She denied the contact history with other seafood, poultry, or livestock. Thus, the patient was diagnosed with erysipeloid based on the clinical manifestation and laboratory findings. Moreover, the antimicrobial susceptibility test suggested that the isolated bacteria are susceptible to imipenem, ciprofloxacin, and piperacillin-tazobactam (Figure S2). Concerning the treatment against *E. rhusiopathiae* infection, piperacillin-tazobactam remained administered for anti-infection. Finally, the patient was discharged from the hospital after her blood culture testing and mNGS were both negative (Day 11). The timeline of the clinical course of the patient was shown in Figure 1C.

**Figure 1.**
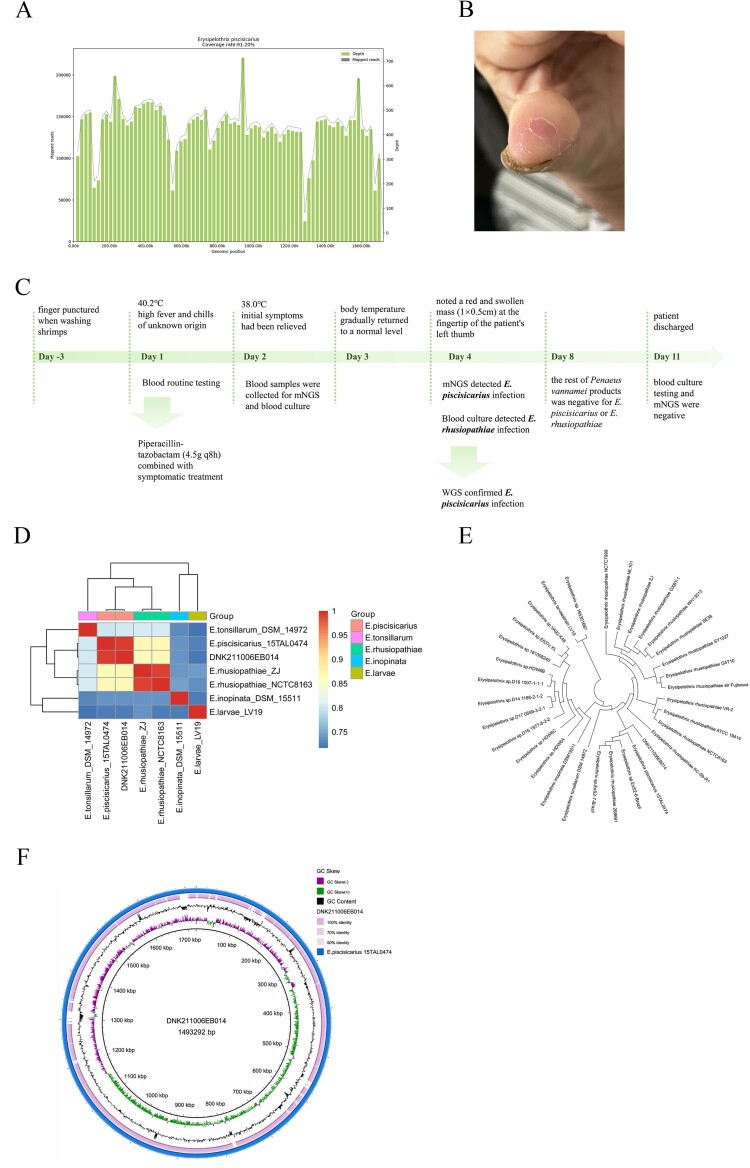
**The diagnosis of *E. piscisicarius* infection and the genome analysis.** (A) The whole genome sequencing yielded a total genome coverage of 91.2%. (B) Red and swollen mass on the patient's left thumb fingertip with a size of about 1 × 0.5 cm. (C) The timeline of the clinical course of the patient. (D) ANI heatmap showed that DNK211006EB014 shared an ANI greater than 0.95 with *E. piscisicarius*_15TAL0474. (E) *E. piscisicarius* is identified by phylogenetic analysis. (F) Circular map of the genome.

To further understand the pathogenicity and virulence of the microorganism (DNK211006EB014) isolated from the patient, genome analysis was conducted. The genome sequence has been deposited in NCBI Sequence Read Archive (SRR19882239, SRR19882240, and SRR19882241). As shown in Figure 1D, DNK211006EB014 shared an average nucleotide identity (ANI) value of 99.41% with *E. piscisicarius* 15TAL0474. The phylogenetic tree further identified the acquired sequence was *E. piscisicarius* (Figure 1E). The assembled genome (Figure 1F) was 1,493,292 bp in length, with a GC content of 37.52%. It contains 1418 genes. 147 virulence-associated genes (VAGs) were detected by comparing in the virulence factors database (VFDB). The prediction revealed that these VAGs encoding the proteins participated in several functional classes, including immune modulation (43), nutritional/metabolic factor (29), adherence (16), exotoxin (11), regulation (11), stress survival (10), effector delivery system (9), biofilm (6), exoenzyme (5), as shown in Table S2. These products are involved in the biosynthesis of capsular polysaccharides (*cps4A*, *cps4H*, *cap8E*, *cap8F*, *cap8G*), and surface protein (*pspA*), closely associated with virulence. All these VAGs were also presented in *E. piscisicarius* 15TAL0474.

## Discussion

We report a human case with *E. piscisicarius* infection through mNGS identification and WGS confirmation. To the best of our knowledge, this is the first case of an *E. piscisicarius* infection that causes severe symptoms in humans.

The patient was punctured in the finger when dealing with *Penaeus vannamei* three days before the onset of the disease, which might be the most probable means of *E. piscisicarius* infection. However, all the specimens of the rest of *Penaeus vannamei* products that remained in the patient's house were negative for *E. piscisicarius* or *E. rhusiopathiae*. It might be interpreted that only a part of the shrimp was infected with the *E. piscisicarius* and the pathogen infected the patient due to the wound caused by these shrimps, which had already been cooked and consumed. For illnesses caused by *E. rhusiopathiae* infection, appropriate antibiotics, adequate debridement, and surgical drainage are the recommended therapies [4]. In this case, we performed the antimicrobial susceptibility test of the isolated bacteria referring to the Clinical and Laboratory Standards Institute (CLSI) criteria for *Erysipelothrix rhusiopathiae*, which indicated that the bacteria are susceptible to piperacillin-tazobactam. Piperacillin is one of the most active agents against *E. rhusiopathiae* infection [5]. Our clinical experience proved that the therapeutic regimen against *E. piscisicarius* is similar to that against *E. rhusiopathiae*.

Culture and culture-based methods have been used to identify clinical pathogens for a long time, however it remains barriers when identifying novel pathogens. In our case, differences arose between the identification results from culture and mass spectrometry and mNGS, considering that the criteria for identification of *E. piscisicarius* through culture are not well established. Compared with the low positive rate and low accuracy of microbiology laboratory cultures [6,7], mNGS contributes to quickly and precisely detect *E. piscisicarius*, suggesting its advantage over the traditional culture in identifying novel pathogens.

It has been reported that capsular polysaccharides [8] and cell surface proteins [9] play essential roles in the virulence of *E. rhusiopathiae*. In our case, 147 VAGs were detected, some of which putatively participated in the biosynthesis of capsular polysaccharide and cell surface protein in *E. piscisicarius*, contributing to its virulence. Considering the scarce information on *E. piscisicarius* in the database, it is suggested to further investigate the pathogenic virulence and antimicrobial resistance.

In conclusion, *E. piscisicarius* can cause human infections and diseases and aquatic animal infections. Once cutaneous lesions are formed after indecent exposure to marine products, it must be paid attention to and treated adequately, and be vigilant to emerging pathogens like *E. piscisicarius*. Compared with conventional methods, mNGS exhibits better detection performance, which is beneficial to clinical practice.

## Supplementary Material

Supplemental Material
