# Supplementary material for: First identification of human infection with Erysipelothrix Piscisicarius by metagenomic next-generation sequencing
Source: Emerg Microbes Infect. 2022 Nov 11;11(1):2781–4. doi: 10.1080/22221751.2022.2140614 (PMC9662008; doi:10.1080/22221751.2022.2140614)
Supplement: Supplemental Material [file TEMI_A_2140614_SM5552.zip › Appendices_revised.docx]

**Technical Appendices**

## Matrix-Assisted Laser Desorption Ionization–Time of Flight (MALDI-TOF) Mass Spectrometry

MALDI-TOF Mass Spectrometry was used to identify the microorganisms isolated from the patient. A bacterial colony was picked up from the blood culture plate after incubation on the medium for 18-24 h at 37℃ and then transferred to a 96-spot polished steel target plate (Bruker Daltonik GmbH, Germany). After dried at room temperature, 1 μl of 70% formic acid (70% v/v) and 1 μl of alpha‐cyano‐4‐hydroxycinnamic acid (HCCA) matrix solution were added to each spot and air-dried separately. The mixture was then inserted into the mass spectrometry system (Bruker microflex LT/SH^TM^, Bruker Daltonik GmbH, Germany) for data acquisition. The spectra were compared with reference libraries for bacterial identification matching.

## Antimicrobial Susceptibility Test

Minimal inhibitory concentrations (MICs) of ciprofloxacin, imipenem, piperacillin-tazobactam, and ceftriaxone to the isolated microorganism were determined using Etest referring to the Clinical and Laboratory Standards Institute (CLSI) criteria for *Erysipelothrix rhusiopathiae*.

## Metagenomic next-generation sequencing (mNGS)

Blood samples were taken from the patients and drawn into 10-mL Vacuumm blood collection tubes (Kang Jian, CHN), and centrifuged to remove cells for reduction of the host-background nucleic acid. cf DNA was extracted using Magnetic Serum/ Plasma DNA Maxi Kit (TIANGEN, CHN) following the manufacturer’s instructions. DNA extraction yield was quantified using a QuantiT dsDNA HS Assay Kit and Qubit 3.0 Fluorometer (Thermo Scientific, USA). Enzymatic shearing was employed for the fragmentation (~200 bp) of DNA molecules and the libraries were then constructed using the Nextera XT DNA Library Preparation Kit (Illumina, USA). The quality of the libraries was assessed by a 2100 Bioanalyzer using the High Sensitivity DNA Assay (Agilent Technologies, USA). Metagenome shotgun sequencing in a single-end 75-bp mode was performed using the NextSeq 500/550 High Output Kit (92 cycles) on an Illumina NextSeq 550 Dx sequencer. The samples as the No-Template Control (NTC) were sequenced simultaneously to assess contaminations during the wet-lab experiments[1, 2].

## Bioinformatics analysis of species-level abundance profiling

Raw sequencing data were first subjected to a quality control process for trimming adapter sequences, removing low-quality tails and reads by Trimmomatic v0.36 [3]. Next, the reads mapping to the human reference genome GRCh37 were excluded using the short-read alignment tool Bowtie v2.2.6 [4]. Read duplication was then performed using in-house scripts. Taxonomic classification of microbial reads was conducted using Kraken v2.0.9-beta [5] and a custom k-mer database that was constructed using 51,543 genomes of ~27,000 species from the NCBI assembly databases [6].

## Whole-genome sequencing (WGS)

Total DNA was extracted using Magnetic Universal Genomic DNA Kit (TIANGEN, China). DNA extraction yield was quantified using a QuantiT dsDNA HS Assay Kit and Qubit 3.0 Fluorometer (Thermo Scientific, USA). Enzymatic shearing was employed for the fragmentation (~200 bp) of DNA molecules and the libraries were then constructed using the Nextera XT DNA Library Preparation Kit (Illumina, USA). The quality of the libraries was assessed by a 2100 Bioanalyzer using the High Sensitivity DNA Assay (Agilent Technologies, USA). Metagenome shotgun sequencing in a single-end 75-bp mode was performed using the NextSeq 500/550 High Output Kit (92 cycles) on an Illumina NextSeq 550 Dx sequencer.

## WGS sequences processing, alignment and analysis

The amplified sequences were assembled and spliced simultaneously with the MEGAHIT v1.2.9 [7] and SPAdes genome assembler v3.14.1 software [8]. Two contigs were obtained and merged, of which those shorter than 500bp were removed and the rest were further spliced with [9]. The acquired sequence was determined to match the *Erysipelothrix piscisicarius* whole genome to evaluate the coverage rate. Subsequently, WGS files of the spices that belong to the *Erysipelothrix* genus available in the GanBank were utilized for annotation. The annotated file was then used for multiple sequence alignment with the Prokka 1.11 software [10]. Species identification was performed with the acquired sequence and the downloaded database file (GCA_003931795.1_ASM393179v1_genomic.fna) through the FastANI software [11].

## Quantitative PCR (qPCR)

Total DNA was extracted using Magnetic Universal Genomic DNA Kit (TIANGEN, China). The concentration and purity of total DNA isolates in the samples were measured spectrophotometrically at wavelengths of A260 and A280. It was performed in a NanoDrop machine (Thermo Scientific, USA). The contents of microbial DNA in the samples were determined with the help of the quantitative PCR method using species-specific primers (sequences: Forward-TGCTGCATCAGCTGTATTTATGG, Reverse-GATCCTTTACAAAGAACCGGATAAT) in a CFX96 thermal cycler (BioRad, China). The procedure of amplification consisted of 40 cycles. Amplification mixture and the reaction procedure are in Table 1 & 2.

Table 1 Amplification mixture of qPCR

| Reagents | Volume (μl) |
| --- | --- |
| Hieff^®^ qPCR SYBR Green Master Mix (High Rox Plus) | 10 |
| Forward Primer (10μM) | 0.4 |
| Reverse Primer (10μM) | 0.4 |
| DNA template | 2 |
| Double-distilled water | 7.2 |
| Total volume (μl) | 20 |

Table 2 The reaction procedure of qPCR

| Steps | Temperature (°C) | Duration | Cycles |
| --- | --- | --- | --- |
| Pre-degeneration | 95 | 5min |  |
| Degeneration | 95 | 10s | 40 |
| Annealing/extending | 60 | 30s |  |
| Melting-curve stage | 95 | 15s |  |
|  | 60 | 1min |  |
|  | 95 | 15s |  |
|  | 95 | 15s |  |

## References:

1. Li M, Yang F, Lu Y, et al. Identification of Enterococcus faecalis in a patient with urinary-tract infection based on metagenomic next-generation sequencing: a case report [Case report]. BMC Infect Dis. 2020;20.

2. Li N, Cai Q, Miao Q, et al. High-Throughput Metagenomics for Identification of Pathogens in the Clinical Settings. Small Methods. 2021;5(1):2000792. doi: https://doi.org/10.1002/smtd.202000792.

3. Bolger AM, Lohse M, Usadel B. Trimmomatic: a flexible trimmer for Illumina sequence data. Bioinformatics. 2014 Aug 1;30(15):2114-20. doi: 10.1093/bioinformatics/btu170. PubMed PMID: 24695404; PubMed Central PMCID: PMCPMC4103590.

4. Langmead B, Salzberg SL. Fast gapped-read alignment with Bowtie 2. Nat Methods. 2012 Mar 4;9(4):357-9. doi: 10.1038/nmeth.1923. PubMed PMID: 22388286; PubMed Central PMCID: PMCPMC3322381.

5. Wood DE, Lu J, Langmead B. Improved metagenomic analysis with Kraken 2. Genome Biol. 2019 Nov 28;20(1):257. doi: 10.1186/s13059-019-1891-0. PubMed PMID: 31779668; PubMed Central PMCID: PMCPMC6883579.

6. Kitts PA, Church DM, Thibaud-Nissen F, et al. Assembly: a resource for assembled genomes at NCBI. Nucleic Acids Res. 2016 Jan 4;44(D1):D73-80. doi: 10.1093/nar/gkv1226. PubMed PMID: 26578580; PubMed Central PMCID: PMCPMC4702866.

7. Li D, Liu CM, Luo R, et al. MEGAHIT: an ultra-fast single-node solution for large and complex metagenomics assembly via succinct de Bruijn graph. Bioinformatics. 2015 May 15;31(10):1674-6. doi: 10.1093/bioinformatics/btv033. PubMed PMID: 25609793.

8. Bankevich A, Nurk S, Antipov D, et al. SPAdes: a new genome assembly algorithm and its applications to single-cell sequencing. J Comput Biol. 2012 May;19(5):455-77. doi: 10.1089/cmb.2012.0021. PubMed PMID: 22506599; PubMed Central PMCID: PMCPMC3342519.

9. Huang X, Madan A. CAP3: A DNA sequence assembly program. Genome Res. 1999 Sep;9(9):868-77. doi: 10.1101/gr.9.9.868. PubMed PMID: 10508846; PubMed Central PMCID: PMCPMC310812.

10. Seemann T. Prokka: rapid prokaryotic genome annotation. Bioinformatics. 2014 Jul 15;30(14):2068-9. doi: 10.1093/bioinformatics/btu153. PubMed PMID: 24642063.

11. Jain C, Rodriguez RL, Phillippy AM, et al. High throughput ANI analysis of 90K prokaryotic genomes reveals clear species boundaries. Nat Commun. 2018 Nov 30;9(1):5114. doi: 10.1038/s41467-018-07641-9. PubMed PMID: 30504855; PubMed Central PMCID: PMCPMC6269478.
