# Supplementary material for: First identification of human infection with Erysipelothrix Piscisicarius by metagenomic next-generation sequencing
Source: Emerg Microbes Infect. 2022 Nov 11;11(1):2781–4. doi: 10.1080/22221751.2022.2140614 (PMC9662008; doi:10.1080/22221751.2022.2140614)
Supplement: Supplemental Material [file TEMI_A_2140614_SM5552.zip › Supplementary files.docx]

**Table S1. Blood routine testing of the patient.**

| c-reaction protein (CRP) concentration | 72.55 mg/L |
| --- | --- |
| neutrophilic leukocyte | 10.27 ×10^9^ cells/L |
| neutrophil ratio | 95.20% |
| lymphocyte ratio | 2.40% |
| monocyte ratio | 2.30% |
| lymphocyte count | 0.25 ×10^9^ cells/L |
| glucose | 9.40 mmol/L |
| standard bicarbonate radical | 26.10 mmol/L |
| pH | 7.529 |
| PaCO_2_ | 28.40 mmHg |
| PaO_2_ | 59.00 mmH |

PaCO_2_, partial pressure of carbon dioxide; PaO_2_, partial pressure of oxygen.

**
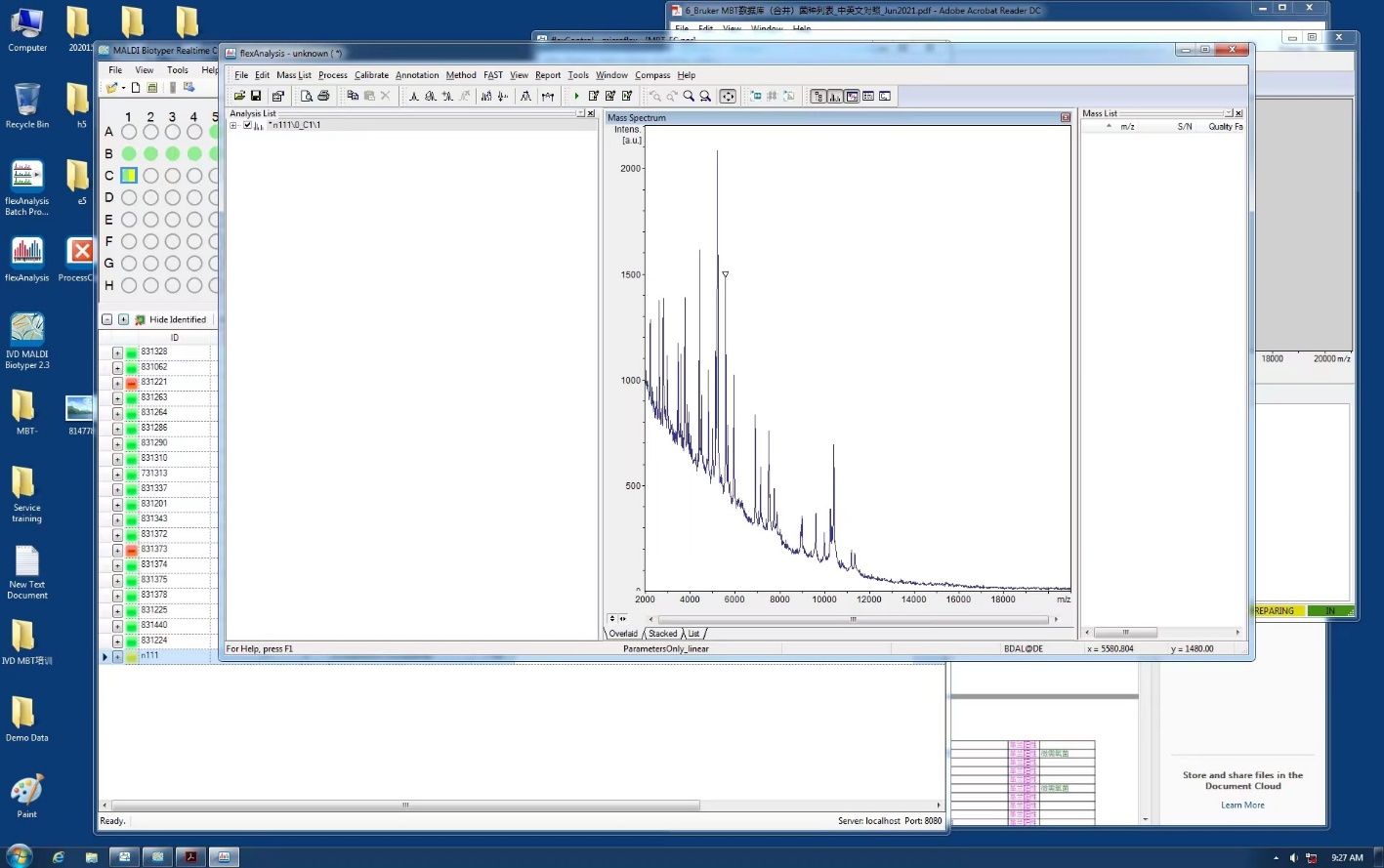
**

**Figure S1. MALDI-TOF mass spectrometry result showed the *E. rhusiopathiae* infection.**

**
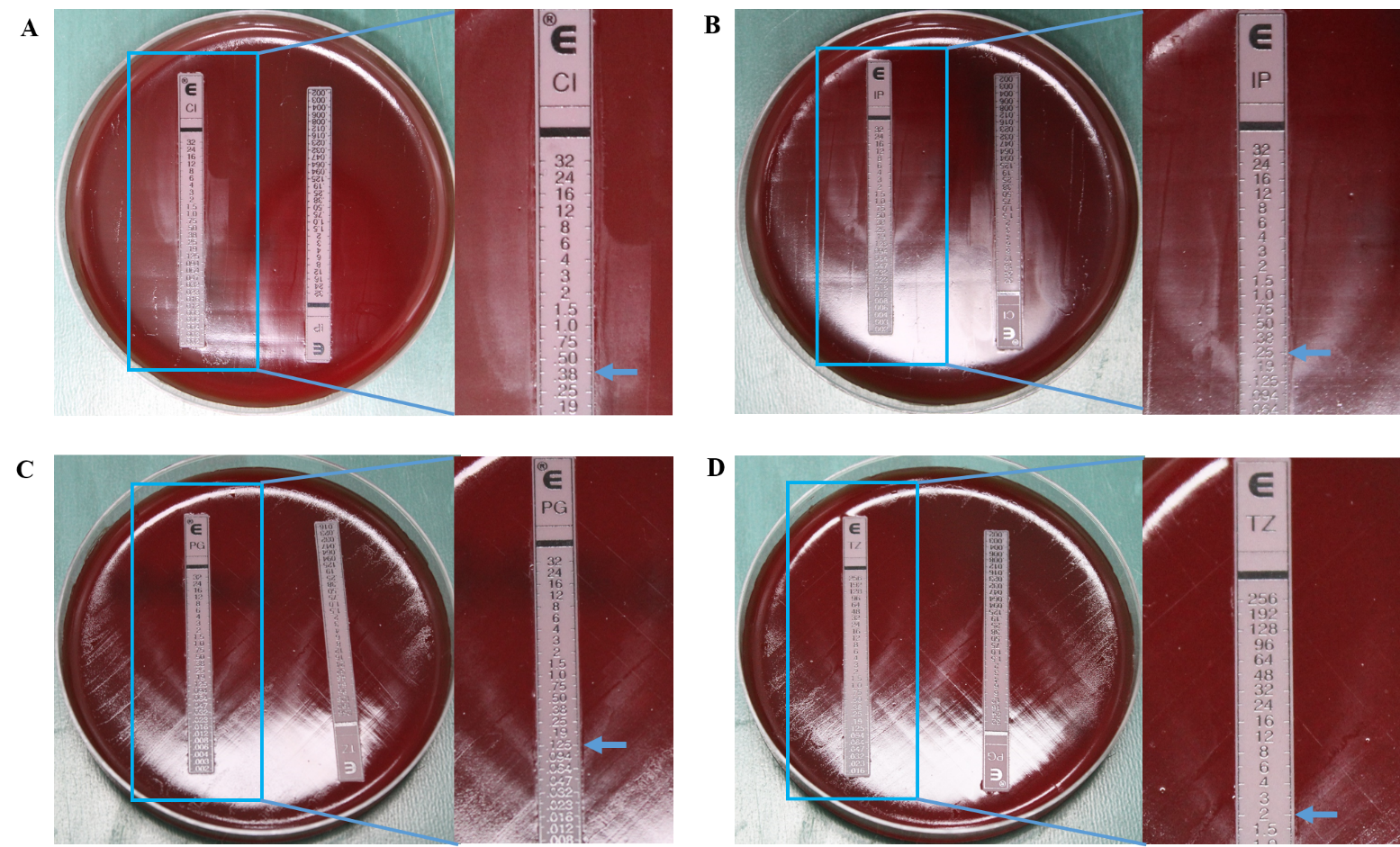
**

**Figure S2. The antimicrobial susceptibility profile of the isolated bacteria. A. CI, ciprofloxacin. The MIC of CI is 0.38 μg/mL. B. IP, imipenem. The MIC of IP is 0.25 μg/mL. C. PG, piperacillin-tazobactam. The MIC of PG is 0.125 μg/mL. D. TZ,** **ceftriaxone. The MIC of TZ is 2 μg/mL. Arrows in blue indicate the MIC values.**

**Table S2. Virulence-associated genes of the genome of DNK211006EB014.**

| #Query_id | Symbol | Functional description from VFDB | Major class |
| --- | --- | --- | --- |
| DNK211006EB014_01398 hypothetical protein | CD0873 | ABC transporter substrate-binding protein | Adherence |
| DNK211006EB014_01399 hypothetical protein | CD0873 | ABC transporter substrate-binding protein | Adherence |
| DNK211006EB014_01165 Putative conjugal transfer protein Rv3659c | VV1_RS15610 | CpaF family protein | Adherence |
| DNK211006EB014_00493 Uncharacterized protein YloA | fbpA | fibronectin-binding protein | Adherence |
| DNK211006EB014_01251 Membrane lipoprotein TpN32 | IlpA | immunogenic lipoprotein A | Adherence |
| DNK211006EB014_01247 High-affinity zinc uptake system binding-protein ZnuA | lmb | laminin-binding surface protein | Adherence |
| DNK211006EB014_01257 Aldehyde-alcohol dehydrogenase | lap | Listeria adhesion protein Lap | Adherence |
| DNK211006EB014_00196 PIII-type proteinase | scpA/scpB | streptococcal C5a peptidase | Adherence |
| DNK211006EB014_01319 Putative conjugal transfer protein Rv3659c | flpF | traffic ATPase | Adherence |
| DNK211006EB014_00421 Putative type II secretion system protein E | pilB | type IV pilus assembly protein PilB | Adherence |
| DNK211006EB014_00528 Tyrosine recombinase XerC | fimB | tyrosine recombinase | Adherence |
| DNK211006EB014_00100 Exodeoxyribonuclease | crc | catabolite repression control protein | Adherence |
| DNK211006EB014_00040 60 kDa chaperonin | groEL | chaperonin GroEL | Adherence |
| DNK211006EB014_01323 Collagen adhesin | acm | collagen adhesin precursor Acm | Adherence |
| DNK211006EB014_00938 Elongation factor Tu | tufA | elongation factor Tu | Adherence |
| DNK211006EB014_00084 D-methionine-binding lipoprotein MetQ | IlpA | immunogenic lipoprotein A | Adherence |
| DNK211006EB014_01139 Carbonic anhydrase 2 | mig-5 | putative carbonic anhydrase | Antimicrobial activity/Competitive advantage |
| DNK211006EB014_01262 Peptidoglycan O-acetyltransferase | algI | alginate o-acetyltransferase AlgI | Biofilm |
| DNK211006EB014_00652 Peptidoglycan O-acetyltransferase | algI | alginate o-acetyltransferase AlgI | Biofilm |
| DNK211006EB014_00012 Serine acetyltransferase | vpsG | exopolysaccharide biosynthesis acetyltransferase VpsG | Biofilm |
| DNK211006EB014_01406 Putative zinc metalloprotease SA1105 | mucP | metalloprotease protease | Biofilm |
| DNK211006EB014_00767 Accessory gene regulator A | fsrA | response regulator | Biofilm |
| DNK211006EB014_00817 Serine protease Do-like HtrA | mucD | serine protease MucD precursor | Biofilm |
| DNK211006EB014_00401 Probable transcriptional regulatory protein YeeN | CBU_1566 | Coxiella Dot/Icm type IVB secretion system translocated effector | Effector delivery system |
| DNK211006EB014_00489 Serine/threonine phosphatase stp | pppA | Pseudomonas protein phosphatase PppA | Effector delivery system |
| DNK211006EB014_00333 V-type ATP synthase alpha chain | vscN | type III secretion system ATPase VscN | Effector delivery system |
| DNK211006EB014_00684 ATP synthase subunit beta, sodium ion specific | yscN | type III secretion system ATPase YscN | Effector delivery system |
| DNK211006EB014_00686 ATP synthase subunit alpha | yscN | type III secretion system ATPase YscN | Effector delivery system |
| DNK211006EB014_00002 Putative bifunctional phosphatase/peptidyl-prolyl cis-trans isomerase | lirB | Dot/Icm type IV secretion system effector LirB | Effector delivery system |
| DNK211006EB014_00793 Peptidyl-prolyl cis-trans isomerase B | lirB | Dot/Icm type IV secretion system effector LirB | Effector delivery system |
| DNK211006EB014_00488 Serine/threonine-protein kinase StkP | ppkA | serine/threonine protein kinase PpkA | Effector delivery system |
| DNK211006EB014_00816 Putative metallo-hydrolase YycJ | yycJ | Zn-dependent hydrolase | Effector delivery system |
| DNK211006EB014_00225 Sialidase A | nanI | exo-alpha-sialidase | Exoenzyme |
| DNK211006EB014_01024 UPF0053 inner membrane protein YtfL | tlyC | hemolysin C | Exoenzyme |
| DNK211006EB014_00291 Hyaluronate lyase | EF3023 | polysaccharide lyase, family 8 | Exoenzyme |
| DNK211006EB014_00974 Xanthan lyase | EF0818 | polysaccharide lyase, family 8 | Exoenzyme |
| DNK211006EB014_01059 Xanthan lyase | EF3023 | polysaccharide lyase, family 8 | Exoenzyme |
| DNK211006EB014_00325 Uncharacterized ABC transporter ATP-binding protein YxlF | cesC | ABC transporter ATP-binding protein, CesC | Exotoxin |
| DNK211006EB014_00623 ABC transporter ATP-binding protein NatA | cesC | ABC transporter ATP-binding protein, CesC | Exotoxin |
| DNK211006EB014_00719 ABC-type transporter ATP-binding protein EcsA | cesC | ABC transporter ATP-binding protein, CesC | Exotoxin |
| DNK211006EB014_00766 ABC-type transporter ATP-binding protein EcsA | cesC | ABC transporter ATP-binding protein, CesC | Exotoxin |
| DNK211006EB014_00321 Leukotoxin export ATP-binding protein LtxB | hlyB | Hemolysin B | Exotoxin |
| DNK211006EB014_01401 Putative hemin import ATP-binding protein HrtA | hlyB | Hemolysin B | Exotoxin |
| DNK211006EB014_00070 ABC transporter ATP-binding protein YtrB | cesC | ABC transporter ATP-binding protein, CesC | Exotoxin |
| DNK211006EB014_00398 ABC transporter ATP-binding protein NatA | cesC | ABC transporter ATP-binding protein, CesC | Exotoxin |
| DNK211006EB014_01269 ABC transporter ATP-binding protein NatA | cesC | ABC transporter ATP-binding protein, CesC | Exotoxin |
| DNK211006EB014_00320 Putative multidrug export ATP-binding/permease protein SA1683 | cyaB | cyclolysin secretion ATP-binding protein | Exotoxin |
| DNK211006EB014_00008 Putative multidrug export ATP-binding/permease protein SA1683 | hlyB | Hemolysin B | Exotoxin |
| DNK211006EB014_00531 Polyisoprenyl-teichoic acid--peptidoglycan teichoic acid transferase TagU | cps4A | capsular polysaccharide biosynthesis protein Cps4A | Immune modulation |
| DNK211006EB014_00713 Biofilm regulatory protein A | cps4A | capsular polysaccharide biosynthesis protein Cps4A | Immune modulation |
| DNK211006EB014_01363 hypothetical protein | licA | choline kinase | Immune modulation |
| DNK211006EB014_01390 hypothetical protein | licB | choline permease | Immune modulation |
| DNK211006EB014_00780 Probable ABC transporter ATP-binding protein HI_0664 | msbA | lipid transporter ATP-binding/permease | Immune modulation |
| DNK211006EB014_01080 Uncharacterized ABC transporter ATP-binding protein TM_0288 | msbA | lipid transporter ATP-binding/permease | Immune modulation |
| DNK211006EB014_01081 Uncharacterized ABC transporter ATP-binding protein Rv1273c | msbA | lipid transporter ATP-binding/permease | Immune modulation |
| DNK211006EB014_01094 Uncharacterized ABC transporter ATP-binding protein TM_0288 | msbA | lipid transporter ATP-binding/permease | Immune modulation |
| DNK211006EB014_01285 Uncharacterized ABC transporter ATP-binding protein Rv1273c | msbA | lipid transporter ATP-binding/permease | Immune modulation |
| DNK211006EB014_01286 Uncharacterized ABC transporter ATP-binding protein TM_0288 | msbA | lipid transporter ATP-binding/permease | Immune modulation |
| DNK211006EB014_00170 Phosphopantetheine adenylyltransferase | kdtB | lipopolysaccharide core biosynthesis protein | Immune modulation |
| DNK211006EB014_00533 UDP-N-acetylenolpyruvoylglucosamine reductase | cpsG | MurB family protein | Immune modulation |
| DNK211006EB014_01196 Peptidoglycan-N-acetylmuramic acid deacetylase PdaC | pdgA | Peptidoglycan N-deacetylase | Immune modulation |
| DNK211006EB014_01391 hypothetical protein | licC | phosphocholine cytidyltransferase | Immune modulation |
| DNK211006EB014_01389 hypothetical protein | licD | phosphocholine transferase | Immune modulation |
| DNK211006EB014_01076 Phosphoglucosamine mutase | cpsG | phosphomannomutase CpsG | Immune modulation |
| DNK211006EB014_00486 Ribulose-phosphate 3-epimerase | rpe | ribulose-phosphate 3-epimerase | Immune modulation |
| DNK211006EB014_00649 hypothetical protein | Cj1416c | sugar nucleotidyltransferase | Immune modulation |
| DNK211006EB014_01149 UDP-N-acetylgalactosamine-undecaprenyl-phosphate N-acetylgalactosaminephosphotransferase | FTT_RS04105 | sugar transferase | Immune modulation |
| DNK211006EB014_01152 UDP-glucose 4-epimerase | cap8E | type 8 capsular polysaccharide synthesis protein Cap8E | Immune modulation |
| DNK211006EB014_01153 UDP-2-acetamido-2,6-beta-L-arabino-hexul-4-ose reductase | cap8F | type 8 capsular polysaccharide synthesis protein Cap8F | Immune modulation |
| DNK211006EB014_01154 UDP-2,3-diacetamido-2,3-dideoxy-D-glucuronate 2-epimerase | cap8G | type 8 capsular polysaccharide synthesis protein Cap8G | Immune modulation |
| DNK211006EB014_00899 Energy-coupling factor transporter ATP-binding protein EcfA1 | FTT_RS04120 | ABC transporter ATP-binding protein | Immune modulation |
| DNK211006EB014_00902 Trifunctional nucleotide phosphoesterase protein YfkN | adsA | Adenosine synthase A | Immune modulation |
| DNK211006EB014_00734 Polyisoprenyl-teichoic acid--peptidoglycan teichoic acid transferase TagU | cps4A | capsular polysaccharide biosynthesis protein Cps4A | Immune modulation |
| DNK211006EB014_01049 Putative glycosyltransferase EpsF | cps4H | capsular polysaccharide biosynthesis protein Cps4H | Immune modulation |
| DNK211006EB014_00554 dITP/XTP pyrophosphatase | orfM | deoxyribonucleotide triphosphate pyrophosphatase | Immune modulation |
| DNK211006EB014_00007 Uncharacterized ABC transporter ATP-binding protein TM_0288 | msbA | lipid transporter ATP-binding/permease | Immune modulation |
| DNK211006EB014_00781 Multidrug resistance ABC transporter ATP-binding and permease protein | msbA | lipid transporter ATP-binding/permease | Immune modulation |
| DNK211006EB014_01121 Uncharacterized ABC transporter ATP-binding protein TM_0288 | msbA | lipid transporter ATP-binding/permease | Immune modulation |
| DNK211006EB014_01122 Uncharacterized ABC transporter ATP-binding protein Rv1273c | msbA | lipid transporter ATP-binding/permease | Immune modulation |
| DNK211006EB014_01115 6-phosphogluconate dehydrogenase, decarboxylating | gndA | NADP-dependent phosphogluconate dehydrogenase | Immune modulation |
| DNK211006EB014_00021 DNA protection during starvation protein 2 | napA | neutrophil activating protein NapA | Immune modulation |
| DNK211006EB014_01407 Phosphatidate cytidylyltransferase | cpsB/cdsA | phosphatidate cytidylyltransferase | Immune modulation |
| DNK211006EB014_00802 hypothetical protein | licD | phosphocholine transferase | Immune modulation |
| DNK211006EB014_00820 Phosphoglucomutase | manB/yhxB | phosphomannomutase | Immune modulation |
| DNK211006EB014_00648 UDP-N-acetylgalactosamine-undecaprenyl-phosphate N-acetylgalactosaminephosphotransferase | FTT_RS04105 | sugar transferase | Immune modulation |
| DNK211006EB014_00647 Putative endo-beta-N-acetylglucosaminidase | pspA | surface protein A | Immune modulation |
| DNK211006EB014_01096 Autolysin | pspA | surface protein A | Immune modulation |
| DNK211006EB014_00001 Teichoic acid poly(glycerol phosphate) polymerase | cpsC | teichoic acid biosynthesis protein, putative | Immune modulation |
| DNK211006EB014_00032 UDP-glucose 4-epimerase | galE | UDP-glucose 4-epimerase | Immune modulation |
| DNK211006EB014_00094 Bifunctional protein GlmU | lpxA/glmU | UDP-N-acetylglucosamine pyrophosphorylase/glucosamine-1-phosphate N-acetyltransferase | Immune modulation |
| DNK211006EB014_01408 Ditrans,polycis-undecaprenyl-diphosphate synthase ((2E,6E)-farnesyl-diphosphate specific) | cpsA/uppS | undecaprenyl diphosphate synthase | Immune modulation |
| DNK211006EB014_00639 Periplasmic zinc-binding protein TroA | lpeA | lipoprotein promoting cell invasion | Invasion |
| DNK211006EB014_00989 5-keto-D-gluconate 5-reductase | flmH | short chain dehydrogenase/reductase family oxidoreductase | Motility |
| DNK211006EB014_00077 Sporulation initiation inhibitor protein Soj | AHML_RS07540 | CobQ/CobB/MinD/ParA family protein | Motility |
| DNK211006EB014_00332 V-type sodium ATPase subunit B | fliI | flagellum-specific ATP synthase | Motility |
| DNK211006EB014_01095 Uncharacterized ABC transporter ATP-binding protein Rv1273c | iroC | ATP binding cassette transporter | Nutritional/Metabolic factor |
| DNK211006EB014_00315 Ferric-anguibactin transport system permease protein FatD | bauD | ferric siderophore ABC transporter, permease protein BauD | Nutritional/Metabolic factor |
| DNK211006EB014_00222 Taurine import ATP-binding protein TauB | hitC | iron(III) ABC transporter, ATP-binding protein | Nutritional/Metabolic factor |
| DNK211006EB014_00436 Phosphate import ATP-binding protein PstB 3 | hitC | iron(III) ABC transporter, ATP-binding protein | Nutritional/Metabolic factor |
| DNK211006EB014_00731 Arginine transport ATP-binding protein ArtM | hitC | iron(III) ABC transporter, ATP-binding protein | Nutritional/Metabolic factor |
| DNK211006EB014_01022 Uncharacterized ABC transporter ATP-binding protein Rv0986 | hitC | iron(III) ABC transporter, ATP-binding protein | Nutritional/Metabolic factor |
| DNK211006EB014_01383 Spermidine/putrescine import ATP-binding protein PotA | fbpC | iron(III) ABC transporter, ATP-binding protein | Nutritional/Metabolic factor |
| DNK211006EB014_01395 sn-glycerol-3-phosphate import ATP-binding protein UgpC | hitC | iron(III) ABC transporter, ATP-binding protein | Nutritional/Metabolic factor |
| DNK211006EB014_00125 Magnesium-transporting ATPase, P-type 1 | mgtB | Mg2+ transport protein | Nutritional/Metabolic factor |
| DNK211006EB014_01346 Calcium-transporting ATPase 1 | mgtB | Mg2+ transport protein | Nutritional/Metabolic factor |
| DNK211006EB014_01265 D-alanine--D-alanyl carrier protein ligase | dhbF | non-ribosomal peptide synthetase, DhbF | Nutritional/Metabolic factor |
| DNK211006EB014_00317 Ferric enterobactin transport ATP-binding protein FepC | bauE | ferric siderophore ABC transporter, ATP-binding protein BauE | Nutritional/Metabolic factor |
| DNK211006EB014_00318 Uncharacterized ABC transporter solute-binding protein YclQ | bauB | ferric siderophore ABC transporter, periplasmic siderophore-binding protein | Nutritional/Metabolic factor |
| DNK211006EB014_00316 Ferric-anguibactin transport system permease protein FatC | bauC | ferric siderophore ABC transporter, permease protein BauC | Nutritional/Metabolic factor |
| DNK211006EB014_00020 Trehalose import ATP-binding protein SugC | hitC | iron(III) ABC transporter, ATP-binding protein | Nutritional/Metabolic factor |
| DNK211006EB014_00036 Macrolide export ATP-binding/permease protein MacB | hitC | iron(III) ABC transporter, ATP-binding protein | Nutritional/Metabolic factor |
| DNK211006EB014_00086 Methionine import ATP-binding protein MetN | fbpC | iron(III) ABC transporter, ATP-binding protein | Nutritional/Metabolic factor |
| DNK211006EB014_00359 Trehalose import ATP-binding protein SugC | hitC | iron(III) ABC transporter, ATP-binding protein | Nutritional/Metabolic factor |
| DNK211006EB014_00378 Glutamine transport ATP-binding protein GlnQ | hitC | iron(III) ABC transporter, ATP-binding protein | Nutritional/Metabolic factor |
| DNK211006EB014_00463 Ribose import ATP-binding protein RbsA | fbpC | iron(III) ABC transporter, ATP-binding protein | Nutritional/Metabolic factor |
| DNK211006EB014_00625 Uncharacterized ABC transporter ATP-binding protein YknY | fbpC | iron(III) ABC transporter, ATP-binding protein | Nutritional/Metabolic factor |
| DNK211006EB014_00846 Cell division ATP-binding protein FtsE | fbpC | iron(III) ABC transporter, ATP-binding protein | Nutritional/Metabolic factor |
| DNK211006EB014_00859 Choline transport ATP-binding protein OpuBA | hitC | iron(III) ABC transporter, ATP-binding protein | Nutritional/Metabolic factor |
| DNK211006EB014_00898 Energy-coupling factor transporter ATP-binding protein EcfA2 | fbpC | iron(III) ABC transporter, ATP-binding protein | Nutritional/Metabolic factor |
| DNK211006EB014_00953 ABC transporter ATP-binding protein YxdL | hitC | iron(III) ABC transporter, ATP-binding protein | Nutritional/Metabolic factor |
| DNK211006EB014_01125 Oligopeptide transport ATP-binding protein OppD | hitC | iron(III) ABC transporter, ATP-binding protein | Nutritional/Metabolic factor |
| DNK211006EB014_01134 Lipoate-protein ligase LplJ | lplA1 | lipoate protein ligase | Nutritional/Metabolic factor |
| DNK211006EB014_00964 Probable cation-transporting ATPase E | mgtB | Mg2+ transport protein | Nutritional/Metabolic factor |
| DNK211006EB014_01043 Oxygen-independent coproporphyrinogen-III oxidase-like protein YqeR | chuW | Putative oxygen independent coproporphyrinogen III oxidase | Nutritional/Metabolic factor |
| DNK211006EB014_00327 Arginine transport ATP-binding protein ArtM | aatC | ATP-binding protein AatC | Others |
| DNK211006EB014_00440 Alkaline phosphatase synthesis sensor protein PhoR | phoR | Possible two component system response sensor kinase membrane associated PhoR | Regulation |
| DNK211006EB014_00441 Alkaline phosphatase synthesis transcriptional regulatory protein PhoP | phoP | Possible two component system response transcriptional positive regulator PhoP | Regulation |
| DNK211006EB014_00546 GTP pyrophosphokinase | relA | Probable GTP pyrophosphokinase RelA (ATP:GTP 3&apos;-pyrophosphotransferase) (PPGPP synthetase I) ((P)PPGPP synthetase) (GTP diphosphokinase) | Regulation |
| DNK211006EB014_01075 Transcriptional regulatory protein CssR | bvrR | response regulator transcription factor | Regulation |
| DNK211006EB014_00427 RNA polymerase sigma factor SigA | sigA/rpoV | RNA polymerase sigma factor SigA | Regulation |
| DNK211006EB014_00379 Signal transduction histidine-protein kinase ArlS | phoR | Possible two component system response sensor kinase membrane associated PhoR | Regulation |
| DNK211006EB014_00380 Transcriptional regulatory protein SrrA | phoP | Possible two component system response transcriptional positive regulator PhoP | Regulation |
| DNK211006EB014_00235 Transcriptional regulatory protein SrrA | mprA | two-component response regulator MrpA | Regulation |
| DNK211006EB014_00238 Transcriptional regulatory protein SrrA | mprA | two-component response regulator MrpA | Regulation |
| DNK211006EB014_00955 Response regulator protein GraR | mprA | two-component response regulator MrpA | Regulation |
| DNK211006EB014_01260 Transcriptional regulatory protein WalR | mprA | two-component response regulator MrpA | Regulation |
| DNK211006EB014_00405 High-affinity zinc uptake system ATP-binding protein ZnuC | mntA | ABC transporter ATP-binding protein MntA | Stress survival |
| DNK211006EB014_00640 Uncharacterized ABC transporter ATP-binding protein HI_1470 | mntA | ABC transporter ATP-binding protein MntA | Stress survival |
| DNK211006EB014_01207 Alkyl hydroperoxide reductase C | ahpC | alkyl hydroperoxide reductase subunit AhpC | Stress survival |
| DNK211006EB014_01297 Uncharacterized protein YxeI | bsh | bile salt hydrolase | Stress survival |
| DNK211006EB014_00366 DNA repair protein RecN | recN | DNA repair protein RecN | Stress survival |
| DNK211006EB014_00582 Chaperone protein ClpB | clpC | endopeptidase Clp ATP-binding chain C | Stress survival |
| DNK211006EB014_00641 Manganese transport system membrane protein MntB | mntB | Manganese transport system membrane protein MntB | Stress survival |
| DNK211006EB014_00114 Superoxide dismutase [Mn] | sodB | superoxide dismutase | Stress survival |
| DNK211006EB014_00234 Peptide methionine sulfoxide reductase MsrB | msrA/BpilB | trifunctional thioredoxin/methionine sulfoxide reductase A/B protein | Stress survival |
| DNK211006EB014_01090 Peptide methionine sulfoxide reductase MsrA | msrA/BpilB | trifunctional thioredoxin/methionine sulfoxide reductase A/B protein | Stress survival |
| DNK211006EB014_00156 Lipoprotein signal peptidase | lspA | signal peptidase II | translational modification |
